# Supplementary material for: Development and Content Validity of the Clinical Needs Assessment for Myalgic Encephalomyelitis (CNAME)
Source: Health Expect. 2026 Aug 26;29(5):e70854. doi: 10.1111/hex.70854 (PMC13518227; doi:10.1111/hex.70854)
Supplement: Supplementary file 1 [file HEX-29-e70854-s001.pdf]

The Clinical Needs Assessment for Myalgic Encephalomyelitis (CNAME)

This questionnaire is a checklist to identify the issues that are important to you and are a priority to address as part of your treatment/management plan.

It has been developed by people with ME/CFS and clinicians working in specialist ME/CFS services and is intended to be used with the other assessment tools in the ME Association's Clinical Assessment Toolkit.

During the development of the CNAME, several aspects of ME/CFS were noted to be so important that nearly everyone highlighted them as a 'need'. So, they have not been included in this final version to minimise the effort of completing the form. However, **you should be aware of them and can expect the service to provide the following:**

- information about the service. For example, what to expect, contact details, and arrangements for support after discharge
- information about ME/CFS in a user-friendly format.
- flexibility to meet your needs. However, please note this may be limited by the resources available within the service.
- staff to be approachable and knowledgeable and to give you the time you need.
- support for your family/carers

The CNAME takes about 10-15 mins to complete in one go, but you can take as long as you want to complete it. If you need help from another person, or another person to complete the CNAME on your behalf, that is fine. If you would prefer a paper copy or complete the survey by phone, or if you have any other questions, please contact the person who sent this form.

There are 21 questions, arranged in two sections: 'Understanding ME/CFS' and 'Care and Support Plan'. Each question has a statement, 'I need ….' And the answer format asks you to agree (that you do have that need) or to disagree (that you do not have that need), or that the need is not important or relevant to you. There are also some open questions that enable you to add for detail if you wish.

SECTION 1: Understanding ME/CFS

1. Are there any accommodations or adjustments that you need to attend the service? Yes/No Add FREE TEXT box to detail the accommodations
   I need …… (answer format = yes / no or not relevant)
2. information and support to explain ME/CFS and my difficulties to my family/ carers/significant others
3. information and support to explain ME/CFS and my difficulties to 'officials' e.g. employers, other health care professionals

4. support to involve my family/ carers/significant others in my care
5. support to build my confidence to manage my illness
6. opportunity to 'meet' others with ME/CFS. This maybe in person, remotely or virtual
7. advice and support about keeping in touch with others, maintaining relationships. or breaking out of social isolation
8. information about ME/CFS support groups/ charities or other resources including advocacy support

Section 2: Care and Support Plan

I need ……..

9. help to work out my needs and priorities and set my own goals
10. support to develop an action plan to address my needs and priorities
11. help to recognise and manage my symptoms
12. information and support about medications e.g. to manage symptoms or other conditions that impact on my ME/CFS. NB. This may include referral or signposting to other services
13. help to identify and manage my limitations including pacing, fluctuations and relapses
14. help (e.g. information, advice, support and/or aids and equipment) with the following activities
    a. basic activities of daily living (e.g. washing, dressing, toileting, eating and drinking)
    b. domestic/ home care (e.g. housework, cooking, gardening, DIY)
    c. family life (e.g. childcare, relationships)
    d. work/ education (e.g. support from employer/ college, reasonable adjustments, occupational health, union support, Access to Work).
    e. benefits and social care (e.g. universal credit, council tax benefit, personal independence payments, blue parking permit)
    f. mobility and 'getting around' including walking or mobility aids (e.g. mobility scooter or wheelchair) and transport (vehicles, public transport)
    g. socialising and leisure
    h. communication including verbal communication, reading, writing, social media
15. Is there anything else you would like us to know about your needs? Add FREE TEXT box

CLINICAL NEEDS ASSESSMENT FOR ME REPORT CARD

This report summarises your clinical needs, which are the things that are a priority for you to work on with the clinical team.

All patients with ME/CFS need the following. You can expect the **service to provide them:**

- information about ME/CFS and the service (e.g. what to expect, contact details and support after discharge).
- flexibility to meet your needs (within the resources available).
- staff to be approachable and knowledgeable, and to give you the time you need.
- support for your family/carers

Your specific needs are summarised below

Needs	Yes	No/ Not relevant
**SECTION 1: Understanding ME/CFS**		
Accommodations to attend the service		
Help to explain ME/CFS and my difficulties to others		
Help to explain ME/CFS and my difficulties to ‘officials’		
Support to involve my family and others in my care		
Support to build my confidence to manage my illness		
Opportunity to ‘meet’ others with ME/CFS.		
Advice about keeping in touch with others, managing/maintaining relationships, or breaking out of social isolation		
Information about ME/CFS support groups/ charities or other resources		
**Section 2: Care and Support Plan**		
Help to work out my needs and priorities and set my own goals		
Support to develop an action plan to address my needs and priorities		
Help to recognise and manage my symptoms		
Information and support about medications		
Help to identify and manage my activity limitations including pacing, fluctuations and relapses		
Help with basic activities of daily		
Help with domestic/ home care		
Help with family life		
Help with work/ education		
Help with benefits and social care		
Help with mobility and ‘getting around’		
Help with socialising and leisure		
Help with communication		
**Comments**		
Accommodations or adjustments to attend the service		
FREE TEXT		
Anything else you would like us to know about your needs		
FREE TEXT		
